# The rationale for primary spine care employing biopsychosocial, stratified and diagnosis-based care-pathways at a chiropractic college public clinic: a literature review

**DOI:** 10.1186/2045-709X-21-19

**Published:** 2013-06-09

**Authors:** Robb Russell

**Affiliations:** 1 Southern California University of Health Sciences, SCU Health System, 16200 E Amber Valley Drive, Whittier, CA 90604, USA

**Keywords:** Low back pain, Diagnosis-based guide, Biopsychosocial, Care-pathways, Primary spine care practitioner

## Abstract

Current management practices for low back pain have led to rising costs without evidence of improvement in the quality of care. Low back pain remains a diagnostic and management challenge for practitioners of many types and is now thought to be a leading global cause of disability. Beyond many published clinical practice guidelines, there are emerging, evidence-based care-pathways including stratification according to the patient's prognosis, classification-based management, diagnosis-based clinical decision guides and biopsychosocial models of care. A proposed solution for successfully addressing low back pain is to train residents at a chiropractic college public clinic to function as primary spine care practitioners, employing evidence-based care-pathways. The rationale for such is described with expected benefits to patient care, improved financial health of medical delivery systems and the training of chiropractors to successfully fill a niche in the healthcare system.

## Introduction

Low back pain (LBP) is one of the most common health complaints, affecting eight out of ten people at some time in their lives [1]. Over the course of one year, 12 -15% of all physician visits in the US will be for a complaint of LBP. Within any three-month period from 2004 – 2008, 24 – 40% of the US population experienced LBP according to self-reported health surveys [2]. The health and economic impacts are not isolated to Western or industrialized countries; it is a global problem [3]. A recent systematic review concluded LBP to be a major problem throughout the world, with the highest prevalence among females and those aged 40–80 years with a point prevalence of 11.9 ± 2.0% and a 1-month prevalence estimated of 23.2 ± 2.9% [4]. According to Haldeman’s review of the Global Burden of Disease Study 2010, LBP is identified as the number-one cause of disability worldwide [5]. LBP is not only prevalent, but it has enormous economic consequences with annual cost estimates in the US ranging from $100 billion on the conservative end to over $600 billion on the upper end [6,7]. Furthermore, fewer than 5% of patients who sustain an episode of LBP each year account for 75% of the total costs [8]. This suggests that all episodes of LBP are not comparable and treating patients with LBP in similar manner is not prudent.

Not only is low back pain prevalent and costly, but it is challenging to diagnose, treat and study [1]. In spite of increased intervention and enormous costs, there has not been an appreciable decrease in the incidence or prevalence of LBP. Deyo *et al*., summed up the problem relative to chronic LBP: “Prescribing yet more imaging, opioids, injections, and operations is not likely to improve outcomes for patients with chronic back pain. We must rethink chronic back pain at fundamental levels [9].”

This paper proposes that any solution for LBP must provide superior clinical outcomes to current practices, create economic value and be based on standardization of care that makes patients, not practitioners, central in the care model. This paper will discuss several models and approaches that may help address these issues. It also outlines how these strategies are being applied at a health science university’s chiropractic college public clinic. Finally, this paper describes the creation of a full-time, chiropractic residency program whose goal is the training of primary spine care practitioners who are intended to be recognized as the primary care professional for the management of LBP.

### Review

Linkage of pain and disability with anatomical abnormality and/or physical impairment, such as might be identified with physical examination procedures or diagnostic studies, is not well supported in the literature. Serious underlying pathologies may account for less than 2% of LBP and defined pain generators no more than 10 to 15% more. This leaves approximately 85% of LBP described as non-specific, mechanical back pain [9]. Those involved with the management of LBP, as noted by Bhangle *et al*., are told that most cases have no recognizable cause and most episodes are not predictable [10]. In a systematic review of clinical guidelines, Dagenais, Caro and Haldeman concluded that no guidelines reported it necessary or even beneficial for practitioners to attempt to identify the anatomical structures involved in LBP after having eliminated potentially serious pathology, specific causes and substantial neurological involvement. It is also suggested that over-ordering diagnostic testing in search of an identifiable pain generator can independently increase the risk of chronicity. In this context, the authors suggest it may be preferable for both practitioner and patients to accept the assessment as a more feasible objective than the diagnosis, which implies a specific pathoanatomic cause that simply cannot be established for the vast majority of patients with low back pain [7].

This view is not without controversy. Murphy and Hurwitz [11,12] claim that a variety of methods exist for detecting many of the causative factors, of which most have known reliability and validity, that help the practitioner identify a relatively specific diagnostic and overall clinical picture of LBP. This approach evolved from evidence correlating: (1) neurophysiological (discogenic and radicular), (2) somatic (*e*.*g*., facet joints, instability, segmental dysfunction and myofascial) and (3) psychological factors that contribute to LBP. Based on these concepts and findings, a diagnosis-based clinical decision can be made that addresses the most important factors for each individual patient and informs the practitioner as to management strategies that have relatively high predictive value of a clinically successful outcome. Although the authors propose a model based on diagnostic classifications, they do not describe it, strictly speaking, to be consistent with the biomedical model as they consider multiple facets, including psychosocial factors, rather than solely focusing on anatomical pathology.

While chiropractors do not contribute to all of the issues raised by Deyo *et al.*[8], there is no room for complacency as the primary methods of treatment employed are no less suspect. Usual care for LBP, from a chiropractic point-of-view, typically includes spinal adjustments or some form of spinal manipulative therapy (SMT). Opinion as to the effectiveness of such treatment is controversial. In spite of some favorable studies, the recommendation for SMT for LBP is often not vigorous. Rather it may be described as good as, no worse than or only slightly better than other treatments [13]. A recent systemic Cochrane-review of SMT for chronic LBP concluded there is high quality evidence that SMT has a statistically significant short-term effect on pain relief and functional status compared to other interventions. There is also varying quality of evidence that SMT has a statistically significant short-term effect on pain relief and functional status when SMT is added to another intervention. However, the effect sizes of SMT were described as small and not apparently clinically relevant [14].

Based on clinical guidelines developed by the American Pain Society and the American College of Physicians, Elder *et al*. state that self-care (patient education, self-care books and patient-structured physical activities) is much less expensive than, and has equivalent or nearly equivalent effectiveness to, costlier interventions such as physical therapy, massage, SMT or acupuncture [15]. Adding confusion to the notion of what constitutes the most effective treatment, Oliveria *et al*. have come to a surprisingly different conclusion, stating there is only moderate quality evidence that self-management of LBP has anything but a small effect on pain and disability [16].

SMT and self-care are cited as examples of treatment methods that may be popular yet are not fully supported by published evidence. There are, of course, many other management options with supporters and detractors or favorable and unfavorable studies. The lack of widely accepted and clearly articulated approaches to successful management of LBP is a source of confusion for healthcare practitioners and patients alike. Haldeman and Dagenais have referred to this as the supermarket approach where consumers and practitioners are confronted with a vast array of possible approaches offered by multiple professions, subspecialists and even commercial product vendors. In this approach, sometimes referred to as provider-centered care, the treatment varies by provider or supplier, rather than being targeted to the patient’s unique needs [17].

There are approaches to the identification and selection of the most efficacious treatment of LBP that eschew the biomedical model and may answer questions as to why a multitude of treatments and countless randomized controlled trials have failed to distinguish a superior approach. These include practice guidelines, biopsychosocial concepts, stratified, classification-based treatment as well as the diagnosis-based clinical decision guide proposed by Murphy and Hurwitz [11]. It would, however, be improper to describe most of these concepts as new.

The spread of clinical guidelines, beginning in the 1960s, represents a regulation of medical care resulting from a confluence of circumstances that mobilized many different groups. Health care was once characterized by individual judgment but public money and large business interests have demanded transparency and regulation. Payers (both private and government) and professional groups wishing to protect their practice-turf began to create guidelines based on discrepancies, institutional and regional, in the incidence of medical and surgical procedures that resulted from observations that neither money nor health care was being distributed wisely. In 1989 the federal United States Agency for Healthcare Research and Quality was created with a mandate to produce practice guidelines [18]. An early attempt in the chiropractic profession includes the so-called Mercy Conference Guidelines [19]. Dagenais, Caro and Haldeman [3] note that there is general agreement among practice guidelines with shared goals when assessing the patient with LBP. These include: (1) sequentially rule out serious spinal pathology, (2) find specific causes of low back pain and (3) find substantial neurologic involvement. Two additional goals to evaluate are: (4) the severity of symptoms and functional limitations as well as (5) identify risk factors for chronicity. Yet, in spite of the publication of numerous guidelines, clinical and economic trends regarding LBP have not improved.

In a 2004 review of the biopsychosocial model, 25 years after it was first described, Borrell-Carrió, Suchman and Epstein [20] state its most enduring contribution to healthcare was to broaden the scope of the clinician’s gaze. The biopsychosocial model, they opine, was a call to change our way of understanding the patient and to expand the domain of medical knowledge to address the needs of each patient. Criticisms of biomedicine are several, with key consideration given to psychosocial variables which are described as more important determinants of susceptibility, severity, and course of illness than had been previously appreciated. Jacob cites opinions that the limitations of the biomedical model became apparent in the late 1970s when it became clear that there was no absolute relationship between tissue damage and the severity of pain experienced [21]. In 2001 Buchbinder *et al*., concluded that “There is now convincing evidence that psychosocial factors, more than biomedical or biomechanical factors, are strongly linked to the transition from acute to chronic back pain disability.” This was based on a successful campaign to alter the beliefs of the general population and medical physicians in Australia, resulting in reduced incidence and cost of workers’ compensation cases compared to controls. Follow-up studies three and four and one-half years after the original campaign found lasting benefits [22-24]. Jacob cautions that those who are unfamiliar with biopsychosocial issues, such as fear-avoidance and fear of movement, may assign blame to a patient for failing to improve rather than understanding the practitioner’s role in effective communication with such a patient [21].

As previously noted, up to 85% of the cases of LBP are described as non-specific and mechanical in nature, essentially categorizing the complaint as a homogeneous entity. Kent and Keating point out, however, that those who employ manual therapy, including chiropractors, believe LBP is heterogeneous and that subcategories of LBP exist. They state that classification-based treatment for LBP has been practiced informally as practitioners make clinical decisions based on clinical experience and pattern recognition [25]. McKenzie long ago made distinctions regarding patient management based on patient characteristics not necessarily related to diagnosis [26]. In 1995 Delitto *et al*., proposed a treatment-based classification approach, acknowledging the pioneering contributions of McKenzie and Cyriax before him [27]. In spite of long-term use, in 2005 Kent and Keating pointed out that such subgrouping of patients had not been subject to clinical validation and considerably more study was required in the field [28].

Recent work suggests that clinically important effects are observed when patients are classified into subgroups and treatment is matched to the patient’s signs and symptoms, rather than basing treatment on a physical diagnosis or providing standardized care to all patients with LBP [29]. Furthermore, recent recommendations from a UK consensus, which included senior researchers experienced in clinical trials for musculoskeletal conditions, recommend including examining subgroups for LBP [30]. Indeed, efforts have been made over the past several years to validate various classification-based treatment schemes although it is generally agreed that these processes are in need of ongoing research [31-33].

Not all studies on classification-based systems find the approach effective for improving outcomes for LBP. A 2012 study by Apeldoorn, Ostelo *et al*., cited possible missing assessments that, had they been employed, might have improved performance of the system. One specific recommendation they identified for future considerations is the inclusion of key psychological factors in the classification [34].

Murphy and Hurwitz cite their approach, the previously described diagnosis-based clinical decision guide, as one that attempts to respond to the challenge of applying the biopsychosocial model and providing individualized treatment programs based on the particular diagnostic features of each patient [11]. Applying these principles in clinical practice they conclude that patients with LBP can be distinguished on the basis of their approach and treatment plans can be formulated based on the diagnosis by utilizing their strategy. They add that their model is practical as it is suitable for use in a clinical practice [35].

A recently validated assessment tool is said to stratify patients with LBP into different treatment categories based on the prognosis for poor clinical outcomes. It does so, in part, by addressing involvement of biopsychosocial predictors of poor outcomes. The screening device, developed at Keele University, England, and known as STarT Back Screening Tool (SBST), consists of a short questionnaire that determines a prognosis, referred to as relatively low, medium or high risk categories for poor outcomes. Patients with LBP who were screened with SBST and treated in the corresponding categories had superior short and long-term disability, quality of life measures and cost savings compared with controls who received treatment considered to be current best practice [36]. Use of this screening tool appears to be suitable in a clinical setting and it may provide important prognostic information for the management of LBP [37].

In a 2012 trial, Wideham, Hill *et al*., evaluated the responsiveness of the SBST to detect clinically meaningful improvement. They found that reductions of at least 3–5 points on the SBST scale correspond to clinically meaningful improvements in patients’ levels of global change, pain severity, disability, pain catastrophizing, fear of movement, and depression. Wideham, Hill *et al*., further concluded that a 3- to 5-point reduction in a patient’s STBT scale translates into a one-risk-category improvement. Importantly, their results suggest that the SBST can be used instead of multiple risk questionnaires to measure recovery from LBP [38].

## Conclusions

These emerging concepts suggest that LBP, specifically that which is typically labeled non-specific and mechanical, is indeed heterogeneous. Emerging models further suggest that by matching prognostic groups, as with SBST [36,38] and/or diagnostic categories as per Murphy and Hurwitz [35], with the proper treatment groups, the probability of successful clinical outcomes is greatly enhanced. It follows that managing LBP in this manner may lead to superior clinical outcomes, reduced disability and reduced healthcare expenditures. In addition, this framework is truly patient-centered rather than the provider-centered, supermarket approach previously described.

A much improved delivery system for LBP, as stated by Haldeman and Dagenais, would be one in which practitioners involved with spine care are knowledgeable about commonly used treatments and therapies and are able to counsel patients on which treatments may be most appropriate for a particular condition [17]. In this vein, a strong case is made by Murphy *et al*., for development of a primary spine care practitioner (PSP) who can fill a niche that serves both to administer care for the majority of patients with LBP, but is also capable of directing patients to other appropriate treatments in the circumstances in which it is warranted. They argue, further, that a PSP who is responsible for front-line diagnosis, management and triage would help achieve meaningful goals of improved clinical outcomes at reduced per capita costs. A PSP, they opine, requires a skill set that includes the ability to apply evidence-based procedures, appropriately educate and motivate patients and effectively prevent and manage disability. They conclude that it is well worth the effort of grooming practitioners toward filling this role [39].

With the knowledge that LBP is often not successfully managed under the prevailing biomedical model and supermarket approach, the implementation of the various components of the aforementioned emerging models may bring meaningful improvements to clinical outcomes, delivery and affordability. As such, a health sciences university, in conjunction with its chiropractic college teaching clinic, has begun offering primary spine care services and a two-year, post-graduate, full-time clinical residency program to develop the skill set of a PSP as identified by Murphy *et al*[39]. The primary spine care services incorporate the SBST in order to help stratify patients into appropriate categories of care, then further classification of treatment and/or referral are patterned after the hospital-based, standardized, spine care pathway, as reported by Paskowski *et al*., [40] and the previously described diagnosis-based clinical decision model of Murphy and Hurwitz [35]. Integration of the skillset necessary to act as a PSP, as described by Murphy *et al*., is essential to the residency program [39].

As the teaching clinic is not a hospital or a medical facility, in order to offer truly patient-centered services, cooperative, integrative alliances have been established with neurosurgeons, interventional pain management specialists, hospital-based physicians and advanced diagnostic testing facilities. The primary spine care services and PSP residency program have attracted the attention of a large medical group that has designated it as their Back Pain Program and directed their 800 community-based primary care and specialist physicians to refer appropriate patients there in lieu of physical therapy, pain management or advanced imaging.

The market for successful care of LBP is enormous, with eight in ten people likely to experience such pain once in a lifetime [1]. It economically prudent for the chiropractic profession and scholastically sensible for a chiropractic college teaching program to offer primary spine care services and a tract for training as a PSP. Acquiring the skills to be a PSP offers those completing such a program the opportunity to distinguish themselves and to function in an integrative yet competitive healthcare marketplace as the primary care professional for the management of LBP. To reiterate the comment of Murphy *et al*., it is, “well worth the effort of grooming practitioners toward filling this role [39].”

The program’s clinical outcomes, patient satisfaction and economic impact will be assessed in an ongoing manner and it is anticipated that, if successful, it will contribute to the future development of more efficacious spine care treatment pathways and management protocol as well as establishing the benchmark of a viable model for training of PSPs to meet the needs of the healthcare market place.

## Competing interests

Robb Russell, D.C. is employed by Southern California University of Health Sciences (SCU) which is comprised of the Los Angeles College of Chiropractic, College of Acupuncture and Oriental Medicine, School of Professional Studies and SCU Health System. He is Executive Director, SCU Health System, Centers of Excellence which consist of Diagnostic Imaging, Sports Medicine, Human Performance and Spine Care as well as their respective postgraduate residency programs. He is Director of Spine Care and the two-year, full-time Spine Care residency program, both of which are described in the accompanying manuscript.
